# Investigating the effects of chiropractic care on resting-state EEG of MCI patients

**DOI:** 10.3389/fnagi.2024.1406664

**Published:** 2024-06-11

**Authors:** Fahimeh Ziloochi, Imran Khan Niazi, Imran Amjad, Alice Cade, Jenna Duehr, Usman Ghani, Kelly Holt, Heidi Haavik, Vahid Shalchyan

**Affiliations:** ^1^Neuroscience & Neuroengineering Research Lab, Biomedical Engineering Department, School of Electrical Engineering, Iran University of Science and Technology, Tehran, Iran; ^2^Centre for Chiropractic Research, New Zealand College of Chiropractic, Auckland, New Zealand; ^3^Faculty of Health & Environmental Sciences, Health & Rehabilitation Research Institute, AUT University, Auckland, New Zealand; ^4^Department of Health Science and Technology, Aalborg University, Aalborg, Denmark; ^5^Riphah International University, Islamabad, Pakistan

**Keywords:** chiropractic, electroencephalography (EEG), mild cognitive impairment (MCI), Alzheimer’s disease, cluster-based permutation test

## Abstract

**Introduction:**

Mild cognitive impairment (MCI) is a stage between health and dementia, with various symptoms including memory, language, and visuospatial impairment. Chiropractic, a manual therapy that seeks to improve the function of the body and spine, has been shown to affect sensorimotor processing, multimodal sensory processing, and mental processing tasks.

**Methods:**

In this paper, the effect of chiropractic intervention on Electroencephalogram (EEG) signals in patients with mild cognitive impairment was investigated. EEG signals from two groups of patients with mild cognitive impairment (*n* = 13 people in each group) were recorded pre- and post-control and chiropractic intervention. A comparison of relative power was done with the support vector machine (SVM) method and non-parametric cluster-based permutation test showing the two groups could be separately identified with high accuracy.

**Results:**

The highest accuracy was obtained in beta2 (25–35 Hz) and theta (4–8 Hz) bands. A comparison of different brain areas with the SVM method showed that the intervention had a greater effect on frontal areas. Also, interhemispheric coherence in all regions increased significantly after the intervention. The results of the Wilcoxon test showed that intrahemispheric coherence changes in frontal-occipital, frontal-temporal and right temporal-occipital regions were significantly different in two groups.

**Discussion:**

Comparison of the results obtained from chiropractic intervention and previous studies shows that chiropractic intervention can have a positive effect on MCI disease and using this method may slow down the progression of mild cognitive impairment to Alzheimer’s disease.

## Introduction

Dementia is a broad group of brain disorders that lead to cognitive impairment, gradual dysfunction, and death of brain cells (Fiscon et al., 2018). Dementia manifests as progressive and persistent deterioration in memory, language, communication, visuospatial skills, personality, or cognition (i.e., judgment, problem-solving, insight, planning and executing tasks, etc.). So far, extensive studies have been conducted to find ways to diagnose and cure these diseases (Orange and Ryan, 2000). Alzheimer’s disease (AD) is one of the most common cognitive disorders and has the highest prevalence of any dementia-type disease (Czigler et al., 2008). Alzheimer’s disease does not have a definitive diagnostic test, and diagnosis is usually one of exclusion (Geldmacher and Whitehouse, 1997). The early diagnosis of AD, or the diagnosis of mild cognitive impairment, is important as early identification can assist in the treatment of people with AD (Gallego-Jutglàa et al., 2015; Faghfouri et al., 2024).

The intermediate stage between normal cognitive impairment due to aging and dementia is defined as mild cognitive impairment (MCI) (Fiscon et al., 2018). There are many symptoms of MCI, but memory loss is one of the risk factors for Alzheimer’s disease (Grundman et al., 2004). Mild cognitive impairment is by its nature and name mild and is considered a precursor to AD. Mild cognitive impairment does not cause serious interference in daily activities, but the rate of its growth and conversion to AD is estimated at 15–20%, while for the typical older adult, this growth rate is around 1–2% (Gallego-Jutglàa et al., 2015).

Mild cognitive impairment may result from changes in the brain that occur in the early stages of AD or other forms of dementia, but the exact cause is still unknown. There is no acceptable evidence that drugs or nutritional supplements are effective in improving cognitive symptoms in patients with mild cognitive impairment (Langa and Levine, 2014). Some evidence shows that regular exercise is effective and beneficial for improving the cognitive symptoms of this disease (Amjad et al., 2018). Previous studies show that one of the important signs of MCI and AD is electroencephalogram (EEG) signal slowing (Dauwels et al., 2010).

Previous research has investigated the effect of chiropractic methods on the function of the nervous system at different levels, for example, motor output, sensory processing, functional performance, and sensorimotor integration (Steven Waterstone et al., 2020). Chiropractic is a method that focuses on the spine and other joints of the body and their relationship with the nervous system. This therapy assesses of disorders in the neuromuscular skeletal system and their effects on the nervous system and the overall health of the body (Gatterman, 2005). Chiropractic intervention in areas of joint dysfunction, most commonly in the spine, can alter the sensory input sent to the brain, altering sensorimotor processing, and effecting subsequent motor functional brain connections (Steven Waterstone et al., 2020).

Over the past decades, a growing body of research has been focused on chiropractic spinal intervention and its effects on the central nervous system (CNS). Research suggests that chiropractic intervention to dysfunctional spinal joints alters the mechanoreceptive input from the spine and that this, in turn, alters how the brain processes, interprets and integrates other interoceptive and exteroceptive information (Steven Waterstone et al., 2020).

In Steven Waterstone et al. (2020) the authors investigated chiropractic spinal manipulation (SM) and its effects on resting-state functional connectivity in 24 subacute to chronic stroke patients monitored by EEG. Functional connectivity of both linear and non-linear coupling was estimated by coherence and phase lag index (PLI), respectively. Results showed a significant increase in functional connectivity from the PLI metric in the alpha band within the default mode network (DMN). The functional connectivity between the posterior cingulate cortex and parahippocampal regions increased following SM. These findings suggest that SM may alter functional connectivity in the brain of stroke patients and highlight the potential of EEG for monitoring neuroplastic changes following SM. In Navid et al. (2020) the authors aimed to evaluate the impact of chiropractic SM on the early somatosensory evoked potentials (SEP) and resting state EEG recorded from chronic stroke patients. Following SM, the N30 amplitude increased by 39%, which was a significant increase compared to the control intervention. The results of this study show that a single session of chiropractic SM increased the amplitude of the N30 SEP peak in a group of chronic stroke patients, which may reflect changes to early sensorimotor function.

In this study, the aim is to investigate the changes in neural activity using the EEG signal pre- and post-chiropractic or control intervention in participants with MCI.

## Methods

### Experimental protocol

The present study used a randomized controlled cross-over design and was conducted at Railway General Hospital in Rawalpindi, Pakistan. The study protocol was approved by the Internal Review Board (approval number IRB-67) at the Atta-ur-Rahman School of Applied Biosciences, National University of Sciences and Technology and Riphah College of Rehabilitation Sciences, Islamabad, Pakistan. The study was also approved by the New Zealand College of Chiropractic Research Committee. The study was conducted following the Declaration of Helsinki.

### Participants

Participants were eligible to participate if they were clinical diagnosis and mini-mental state examination (MMSE) scores were between 19–24 as assessed by physicians. The participants were ineligible to participate if they showed no evidence of spinal dysfunction, had absolute contraindications to spinal manipulation (e.g., spinal fracture, atlantoaxial instability, spinal infection, spinal tumor, or cauda equina syndrome), or previously experienced a serious adverse event following chiropractic spinal manipulation.

26 individuals with MCI aged 63 ± 5 years (4 Females) were randomly divided into control and chiropractic groups (13 subjects in each group); the subjects gave their written informed consent to participate in the study.

### Experimental protocol

The EEG was recorded (2 min) at a sampling rate of 2048 Hz from 62 channels using a REFA amplifier (TMSi, Twente, The Netherlands) according to the 10–20 electrode system. The ground electrode was placed at AFz. The impedance of the electrodes was kept below 10 kΩ. The subjects were asked to focus on a white fixation cross with a black background displayed in the center of a computer screen while minimizing eye blinks, eye movements, and facial movements.

### Interventions

The chiropractic SM and control interventions were similar to those used in previous studies that investigated the neurophysiological effects of chiropractic SM (Haavik and Murphy, 2012; Niazi et al., 2015; Lelic et al., 2016; Holt et al., 2019; Navid et al., 2019a, 2020; Steven Waterstone et al., 2020; Navid et al., 2022). The same chiropractor, a graduate of the New Zealand College of Chiropractic, with over five years’ of experience, performed the experimental and control interventions. At the end of the second session, the subjects were asked if they had perceived that they had undergone active treatment (‘yes’ or ‘no’).

### Chiropractic spinal manipulation

The New Zealand registered chiropractor manually performed high-velocity, low-amplitude manipulations or via instrument assistance to the spine or pelvic joints, representing typical treatments used in the chiropractic profession (Cooperstein and Gleberzon, 2004). The chiropractor selected site(s) for SM based on routinely used clinical indicators (Triano et al., 2013), which included tenderness to palpation of the relevant joints; restricted intersegmental range of movement on manual palpation; palpable asymmetric intervertebral muscle tension, and any unusual or blocked joint play and end-feel of the joints. Chiropractic SM was applied at multiple levels of the spine and pelvis depending on each patient’s clinical findings. Chiropractic SM was provided where clinically warranted and was either manual, high-velocity, low-amplitude thrusts, or instrument-assisted thrusts to the spine or pelvic joints. Each participant received adjustments at various levels of the spine, as deemed suitable following the chiropractic examination. The duration of each chiropractic visit averaged around 15 min, during which no additional interventions were administered by the chiropractor.

### Control intervention

The control intervention acted as a physiological control for possible changes occurring due to the cutaneous, muscular, or vestibular input that would have occurred with the movements involved in preparing a patient for chiropractic SM. The chiropractor applied a simulated (sham) SM by passively moving the subject’s head, spine, and body into positions approaching those used in the chiropractic manipulation intervention group. However, the chiropractor took care to not provide a manipulative thrust/impulse or to take a spinal motion segment to the end-range tension.

In this study, EEG data of 26 patients with MCI, who were randomly divided into control and chiropractic groups (13 subjects in each group), were analyzed. The average age of the chiropractic group is 66 and the average age of the control group is 65.

All subjects were subjected to a clinical diagnosis and mini-mental state examination (MMSE) scores were assessed by physicians. Subjects were asked to sit still and avoid any movement of hands, fingers, or facial twitches (blinking). Two minutes of EEG data were recorded for each person pre- and post-intervention.

### Data processing

In this study, data preprocessing has been done using MATLAB software and EEGLAB toolbox. One of the common statistical approaches to removing the artifact is the independent component analysis (ICA) method (Luck, 2014). The ICA is a method to separate the signal into the sum of several other signals so that the resulting signals are independent and have a non-Gaussian distribution. In this study, the EEG signal was pre-processed to remove artifacts caused by eye and body movements using the ICA method. After using ICA, the average reference was calculated for the data. Then, using EEGLAB, the data was filtered with a finite impulse response (FIR) filter with a Kaiser window (*β* = 5.653) of order 7420 (corresponding to a transmission bandwidth of 1 Hz) in the range of 0.5 to 40 Hz. Also, the sampling rate was reduced to 512 Hz (Navid et al., 2019b).

To obtain an artifact-free signal, the removal of epochs with artifacts was done using the following four criteria and with the help of ERPLAB: (1) Voltage greater than 100 microvolts. (2) Peak-to-peak voltage greater than 150 microvolts with a window of 200 milliseconds and a step of 100 milliseconds. Peak-to-peak voltage is the difference between the most positive and the most negative voltage in a window. (3) Voltage greater than 100 microvolts from the step function with a window of 200 milliseconds and a step of 50 milliseconds. This step function was used to find quasi-step changes in voltage during saccadic eye movements as well as blinking. (4) A difference between two consecutive samples of more than 50 microvolts, is used to detect sudden shifts in voltage between two consecutive sample (Lopez-Calderon and Luck, 2014).

### Nonparametric cluster-based permutation

EEG data has a spatio-temporal structure where the signal consists of multiple electrode locations and time points determined by the sampling frequency. Furthermore, when investigating the effect of an intervention, data are collected in two test conditions: pre- and post-intervention. Multiple comparisons, assessing for a difference between the two conditions, using statistical methods such as cluster-based permutation tests (Oostenveld and Maris, 2007). In this study, 5,000 permutations were performed (Kajihara et al., 2015). To check the significance probability of clusters, the Monte Carlo method was used, because the permutation distribution can be estimated using this method (Ernst, 2004). With the help of this method, the *p*-value is obtained. If the *p*-value is lower than the critical alpha level (usually 0.05), it can be concluded that the data related to different test conditions are significantly different (Oostenveld and Maris, 2007). The analysis was done in MATLAB software using the FieldTrip toolbox (Oostenveld et al., 2011). First, the pre-processed data were loaded into MATLAB software. Then frequency analysis was done to estimate the power spectrum. Power estimation for this study was done using non-parametric spectral estimation and a single Hanning taper. The purpose of the non-parametric cluster-based permutation test was to determine the statistical difference between EEG measurements before and after the intervention.

The relative power was calculated for each of the frequency bands and then a permutation test was performed to check the statistical differences pre- and post-intervention in two chiropractic and control groups. Finally, the results were shown as topographic maps. The statistical difference was investigated in six bands: delta (4–0.5), theta (4–8), alpha1 (8–11), alpha2 (11–14), beta1 (14–25) and beta2 (25–35).

### SVM classification

There are many classifiers, such as linear discriminant analysis (LDA), support vector machine (SVM), artificial neural network (ANN), and Bayesian classifier that have been effectively used in medical and other fields. Among these, SVM is widely used to classify neurological and brain disorders such as epilepsy and AD (Sharma et al., 2018). A support vector machine was developed by Vapnik and Cortes for a two-class classification problem (Cortes and Vapnik, 1995).

**Figure 1:**
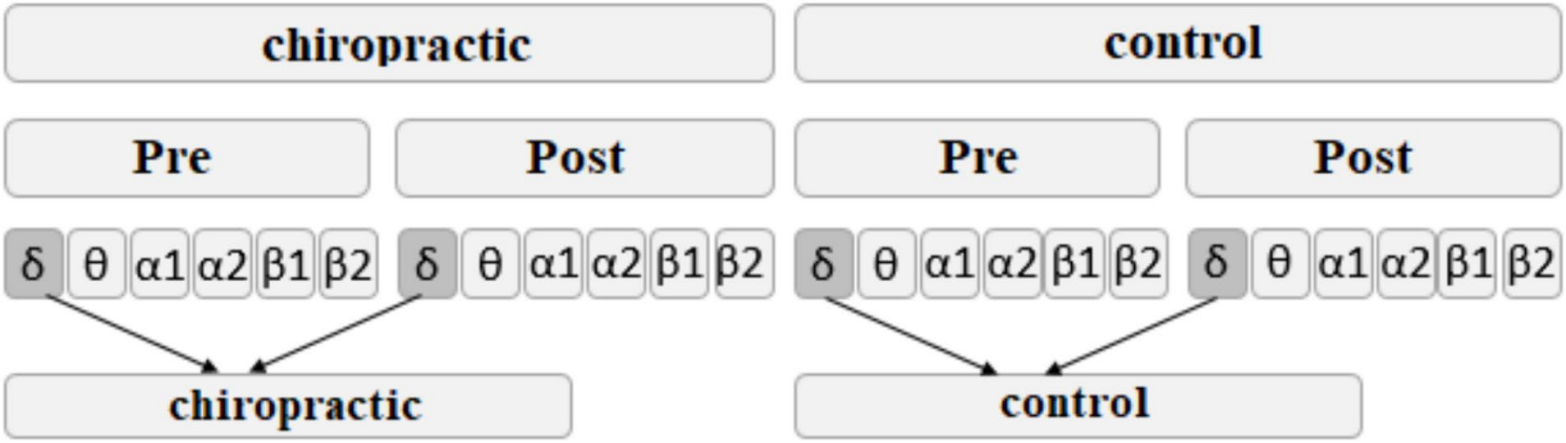
The process of creating features for classification with SVM. This process was repeated for each of the frequency bands.

In order to check whether the difference between pre- and post-measurements was different between the chiropractic or control interventions and also to find the channels with more power change after the intervention, we used the SVM method. The relative power difference before and after the intervention was used as a feature in the classification. First, the relative power of each of the frequency bands was calculated for the signals pre- and post the interventions, creating six data sets each corresponding to one of the frequency bands. For each band, the relative power before the intervention was subtracted from the relative power after the intervention. This process was repeated for each participant. Figure 1 shows the process of creating features for classification. To increase the number of samples, 5 s intervals were selected. In this way, 24 samples were created for each participant and a total of 312 samples were created for each group.

The classification performance in different frequency bands was obtained using the accuracy criterion, according to the following equation, where tp is true positive, tn is true negative, fp is false positive, and fn is false negative.


(1) $$\mathrm{Accuracy}=\frac{tp+tn}{tp+fp+tn+fn}\times 100\%$$

High accuracy shows that the classifier can distinguish the features related to the chiropractic group from the features related to the control group. In other words, the high accuracy shows that the differences that occur after applying the chiropractic method are different from the changes after a control intervention.

### Coherence analysis

EEG coherence can be defined as the normalized cross-power spectrum per frequency of two signals recorded simultaneously at different sites of the scalp. It is a measure of the synchronization between the two signals and may be interpreted as an expression of their functional interaction (Locatelli et al., 1998). The coherence estimates represent the temporal synchronization or functional coupling of the two cortical populations generating the scalp EEG data collected by the paired electrode (Moretti et al., 2008). In order to investigate the effect of the intervention on EEG signal synchronization, the coherence between different areas was calculated. Right and left frontal, right and left parietal, right and left temporal, and occipital regions were considered. To obtain the coherence between the two regions, first the coherence of all pairs of channels in the two regions was calculated and then averaged. In order to increase the number of samples, coherence was calculated in 5 s intervals. In this way, for each subject, the coherence of 24 intervals of 5 s (120 s in total) was obtained. For statistical comparison, Wilcoxon rank sum test was used to compare pre and post coherence and to compare the coherence differences in the control and chiropractic groups.

## Results

In the study evaluating the effectiveness of subject blinding, out of 26 participants, three suspected that one of their sessions was inactive. Among these three, just one correctly identified the order of the interventions (chiropractic or control) they received.

### Nonparametric cluster-based permutation

**Figure 2:**
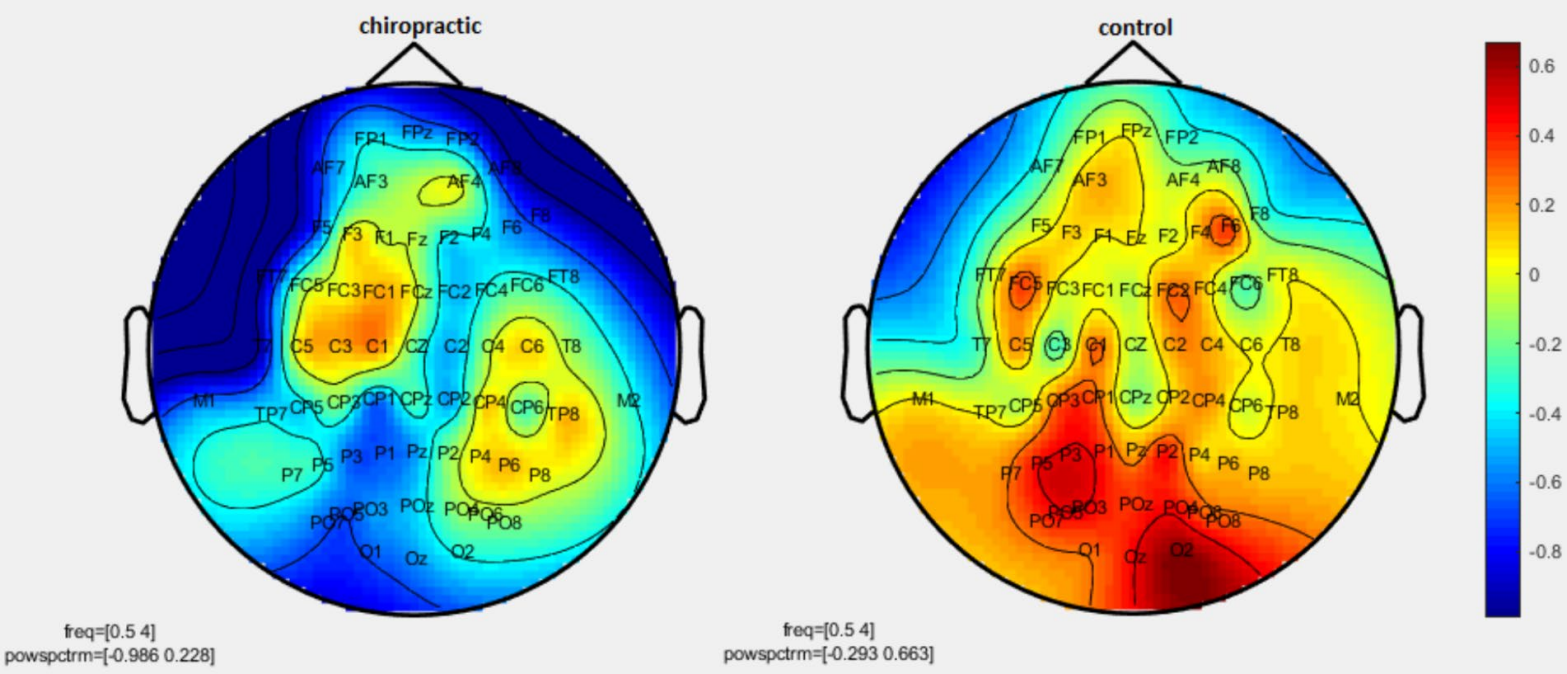
Topographic difference maps in comparison of the pre-and-post for control and chiropractic groups in the delta frequency band (0.5–4 Hz).

**Figure 3:**
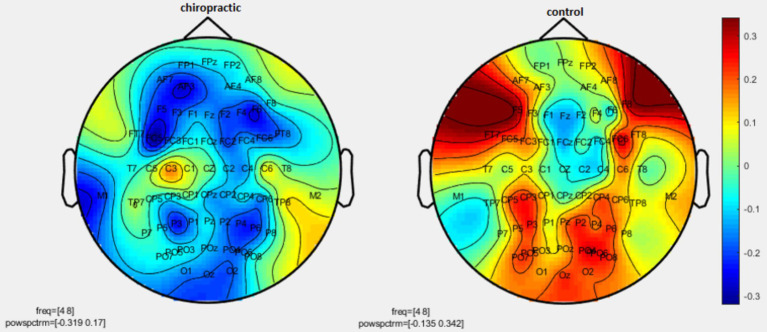
Topographic difference maps in comparison of the pre-and-post for control and chiropractic groups in theta frequency band (4–8 Hz).

**Figure 4:**
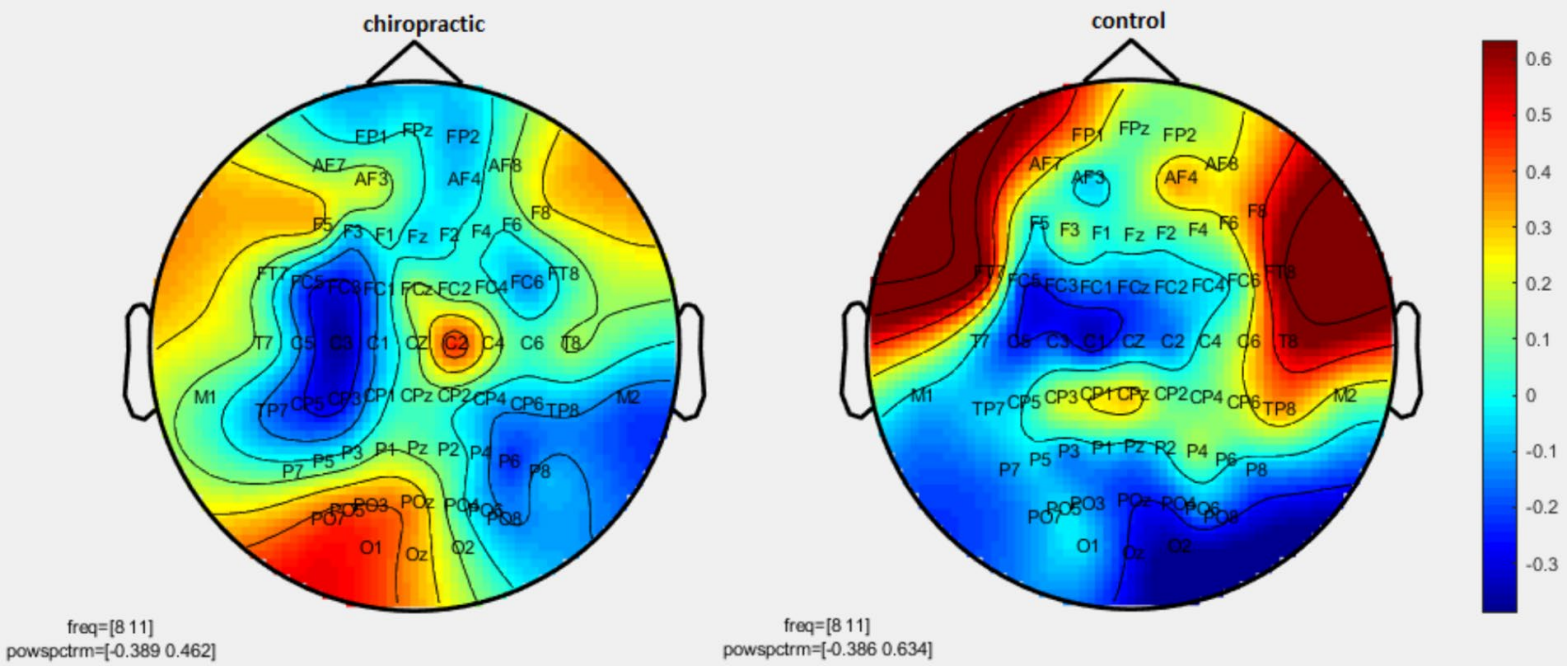
Topographic difference maps in comparison of the pre-and-post for control and chiropractic groups in the alpha1 frequency band (8–11 Hz).

**Figure 5:**
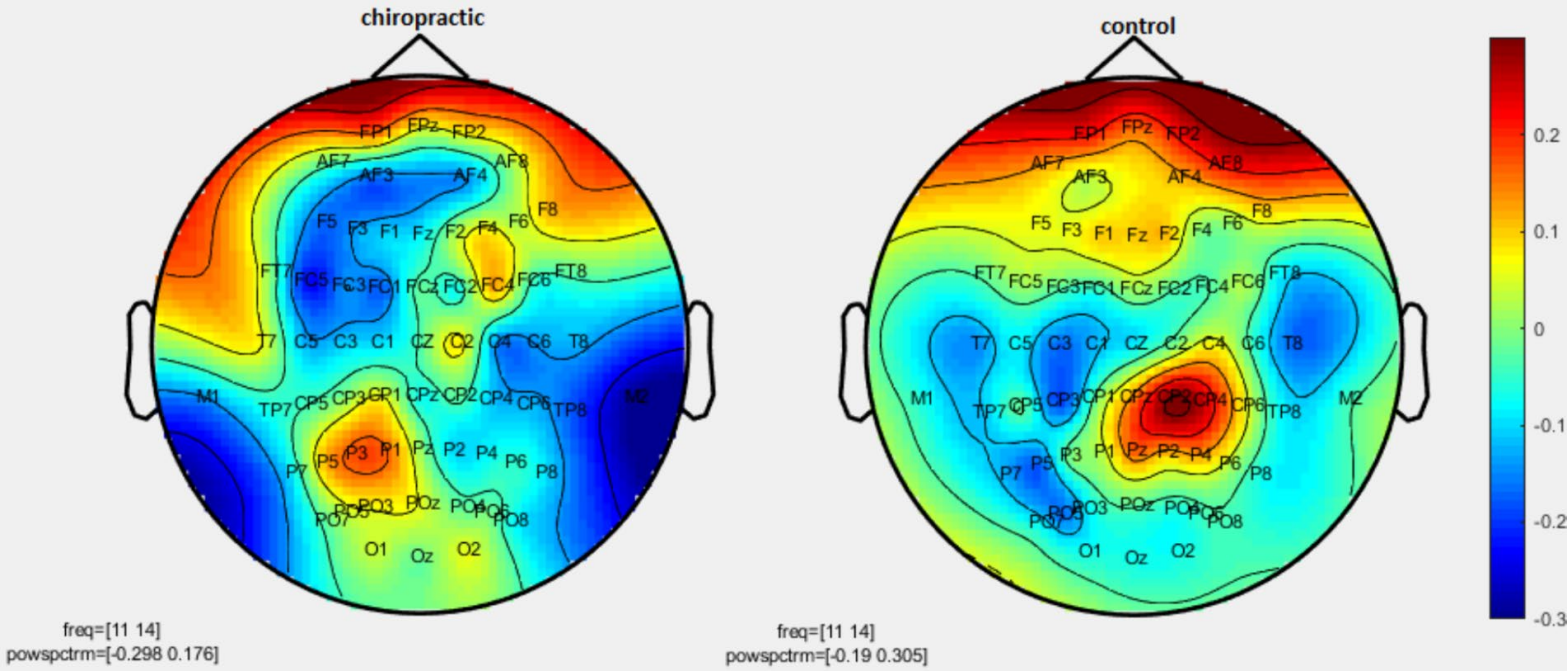
Topographic difference maps in comparison of pre-and-post for control and chiropractic groups in the alpha2 frequency band (11–14 Hz).

**Figure 6:**
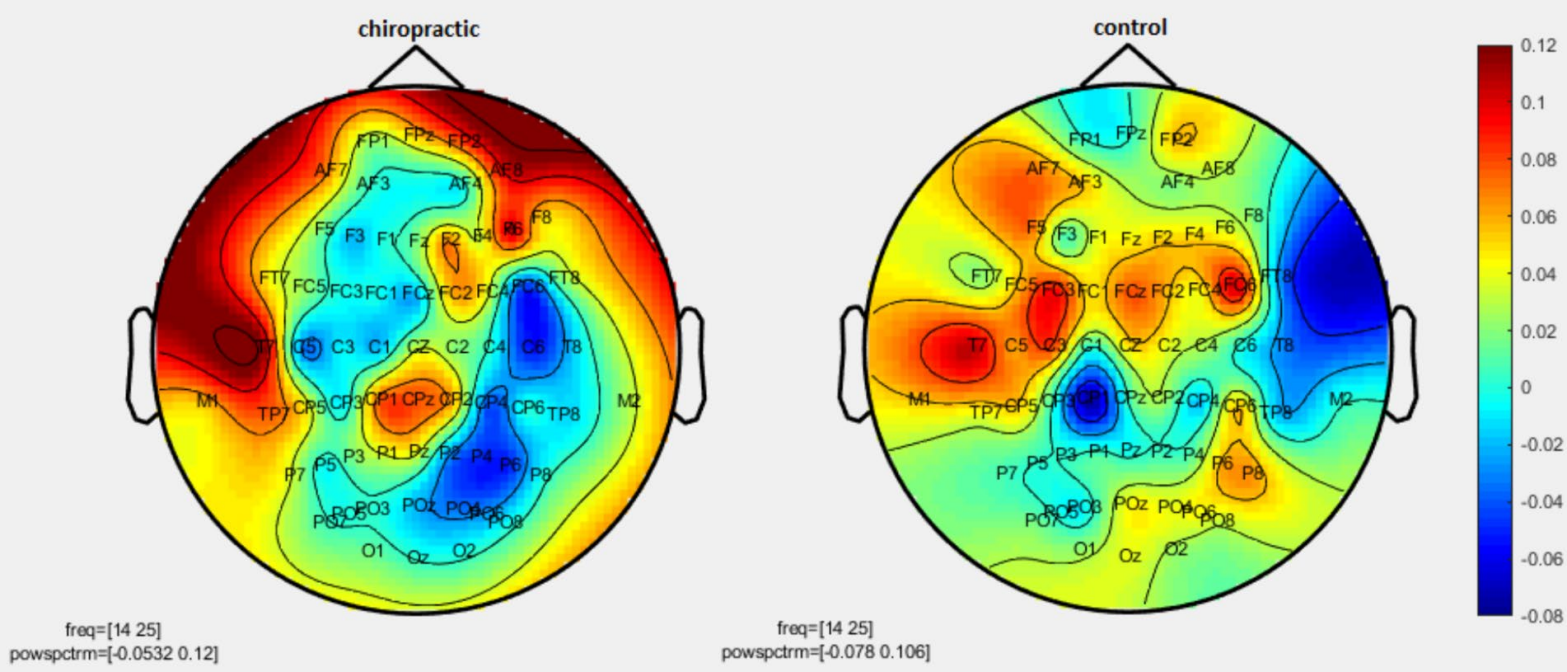
Topographic difference maps in comparison of the pre-and-post for control and chiropractic groups in the beta1 frequency band (14–25 Hz).

**Figure 7:**
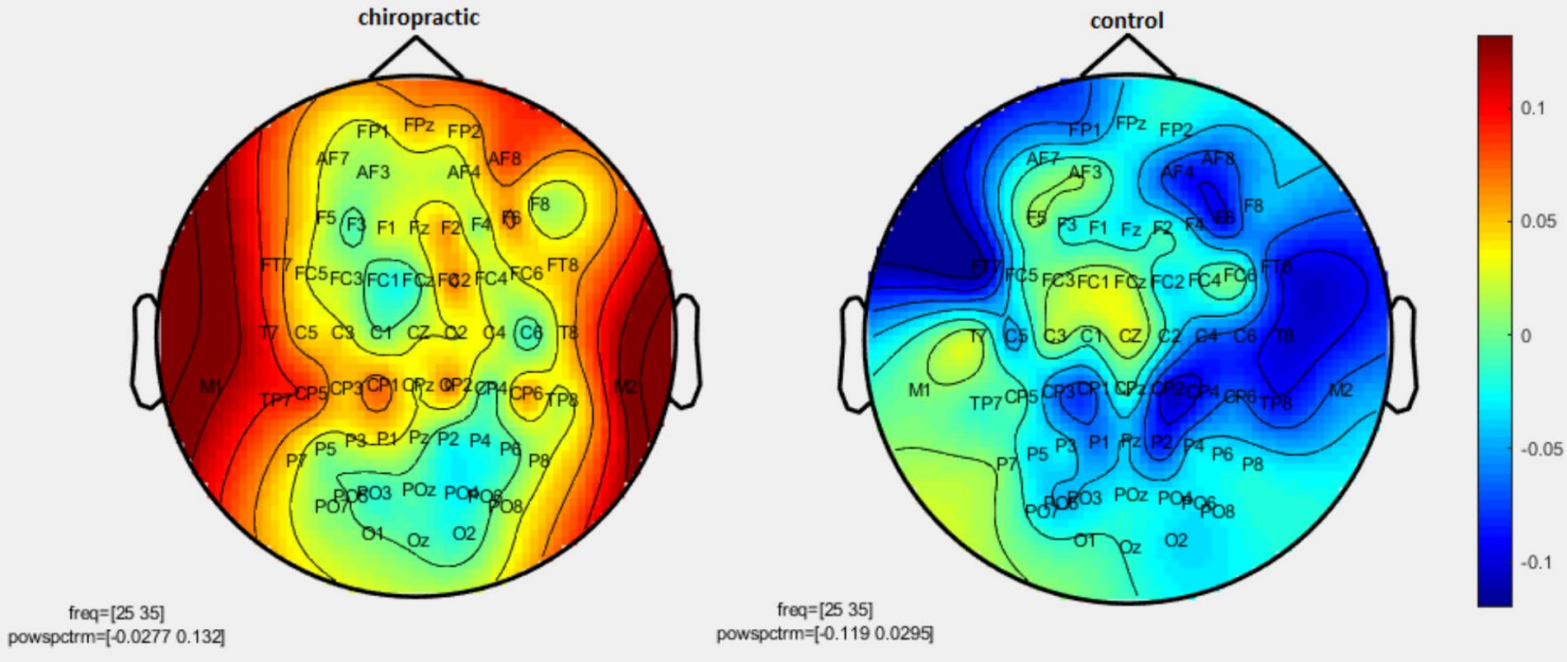
Topographic difference maps in comparison of the pre-and-post for control and chiropractic groups in the beta2 frequency band (25–35 Hz).

Permutation tests were performed to investigate the statistical difference in the power spectrum for two comparisons: (1) pre-measurements for control vs. post-measurements for control, and (2) pre-measurements for spinal manipulation vs. post-measurements for chiropractic intervention. *p*-values were obtained for significant differences and the results were visualized as topographic maps. These topographic maps show the different values for each channel. Figures 2–7 show the mean difference of relative power between pre- and post-conditions in each frequency band in the control and chiropractic groups. In the chiropractic group, a decrease in power was observed for the delta (0.5–4 Hz) and theta (4–8 Hz) bands, and an increase in power was observed for the beta2 band (25–35 Hz) post the intervention.

Data analysis using nonparametric cluster-based permutation resulted in *p*-values for cluster changes. Clusters were identified in delta and theta for the chiropractic and beta2 in the control group. Based on the *p*-values for each cluster no statistical significance was found in the two comparisons. Clusters identified in each frequency band and the *p*-value of each are listed in Table 1.

**Table 1:** Results from the nonparametric permutation test.

Frequency band	Chiropractic		Control	
	Clusters	*p*-value	Clusters	*p*-value
Delta (0.5–4 Hz)	3	0.1506–0.1673–0.1712	–	–
Theta (4–8 Hz)	2	0.1702–0.1854	–	–
Alpha1 (8–11 Hz)	–	–	–	–
Alpha2 (11–14 Hz)	–	–	–	–
Beta1 (14–25 Hz)	–	–	–	–
Beta2 (25–35 Hz)	–	–	1	0.1656

Clusters were identified in delta and theta bands in the chiropractic group and the beta2 band in the control group. Based on the *p*-values for each cluster no statistical significance was found.

### SVM classification

The purpose of using SVM was to investigate whether this method could complement the results obtained from the statistical method. The process of training and testing the classifier was repeated 20 times in each run, 70% of the subjects were randomly selected for training and 30% for testing. The mean accuracy in 20 runs was considered as the final accuracy.

For all frequency bands, the classifier separated the control and chiropractic groups with high accuracy. The highest accuracy was obtained in beta 2, theta, and delta bands, respectively. In the permutation test, the clusters were found in these frequency bands (Table 2).

**Table 2:** Results from the SVM classifier in the classification of control and chiropractic groups.

Frequency band	Delta	Theta	Alpha1	Alpha2	Beta1	Beta2
Accuracy	90.55	95.26	82.71	80.96	89.66	99.74

In this case, the features from all channels are used for classification.

**Figure 8:**
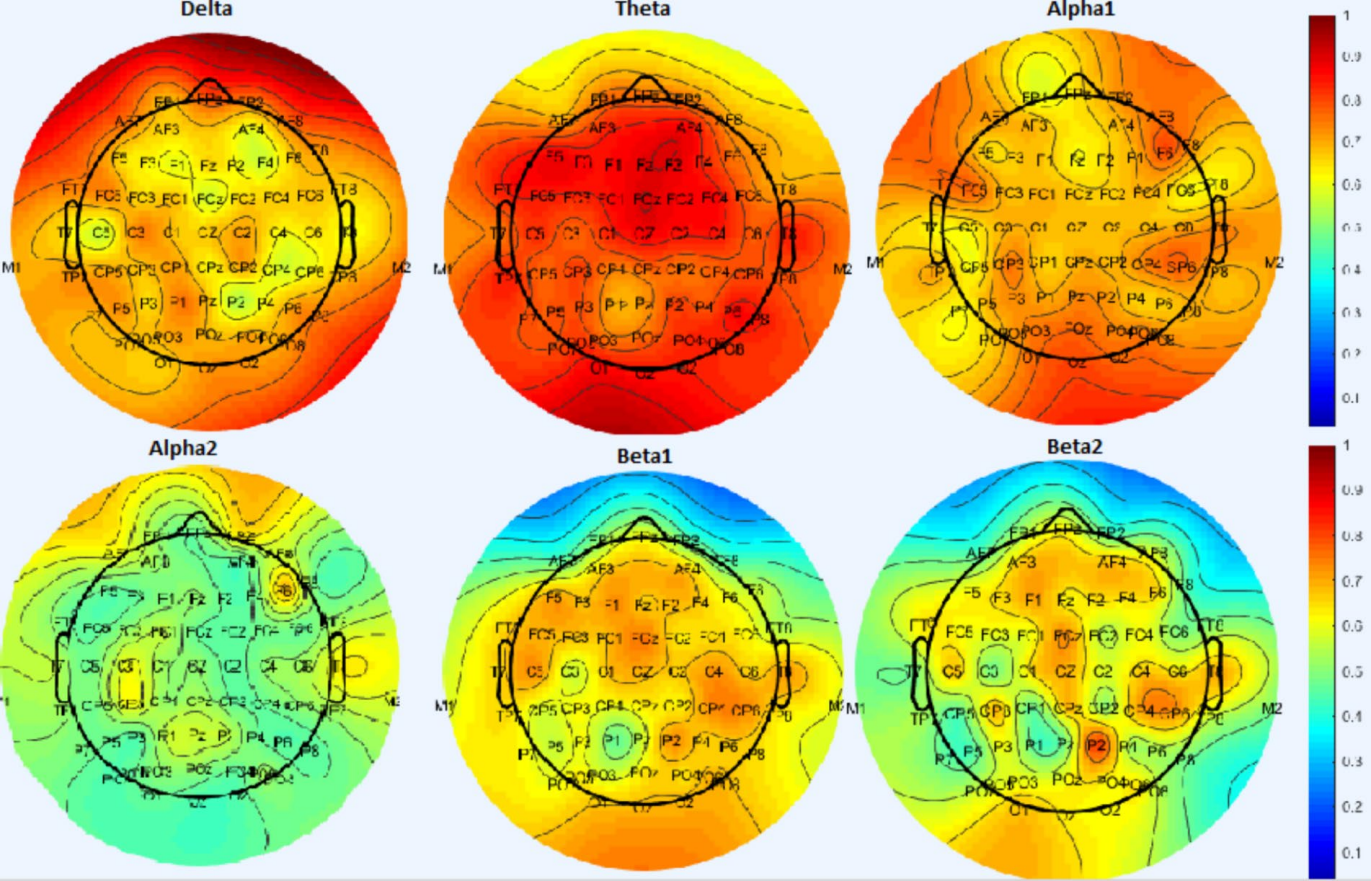
Results from the SVM classifier in the classification of control and chiropractic groups for each channel. Warm colors show more accuracy in classification.

The SVM method was used to investigate which areas had the most impact in the intervention. In this case, the relative power difference pre- and post-intervention in each frequency band was selected as a feature and each time the data related to a channel was considered separately as a feature. The accuracy obtained for each channel in the classification of control and chiropractic groups is shown in Figure 8.

In the delta band the highest accuracy is related to the frontal area (AF8, AF7, and FPz channels), in theta band the highest accuracy is related to the frontal, central, and occipital areas (F5-F3-F1-Fz-F2-FC1-FCz-FC2-Fc4 channels), In alpha1 band, the highest accuracy is related to the occipital (Oz) and right frontal channels (F6), in alpha2 band, the highest accuracy is related to the right frontal and temporal areas (T8, F6), in the beta band, the highest accuracy is related to the frontal channels (AF3-AF4-F3-F1-F4-FCz-Cz) and right temporal lobe (T8-C6-CP6) and parietal (P2) which is higher in beta 2 than beta 1.

### Coherence analysis

**Figure 9:**
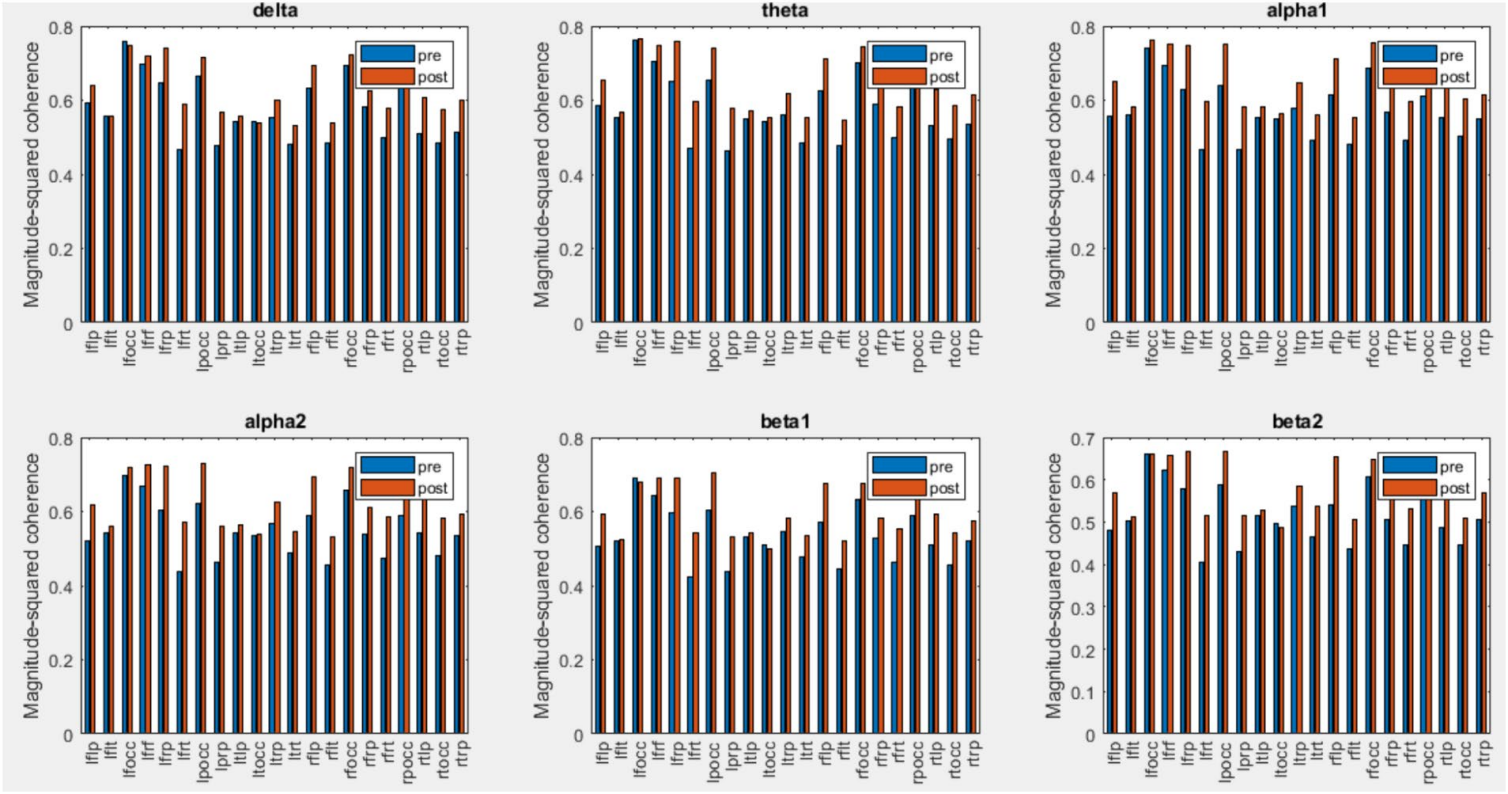
Mean coherence values of all subjects for pre and post-intervention in the chiropractic group.

**Figure 10:**
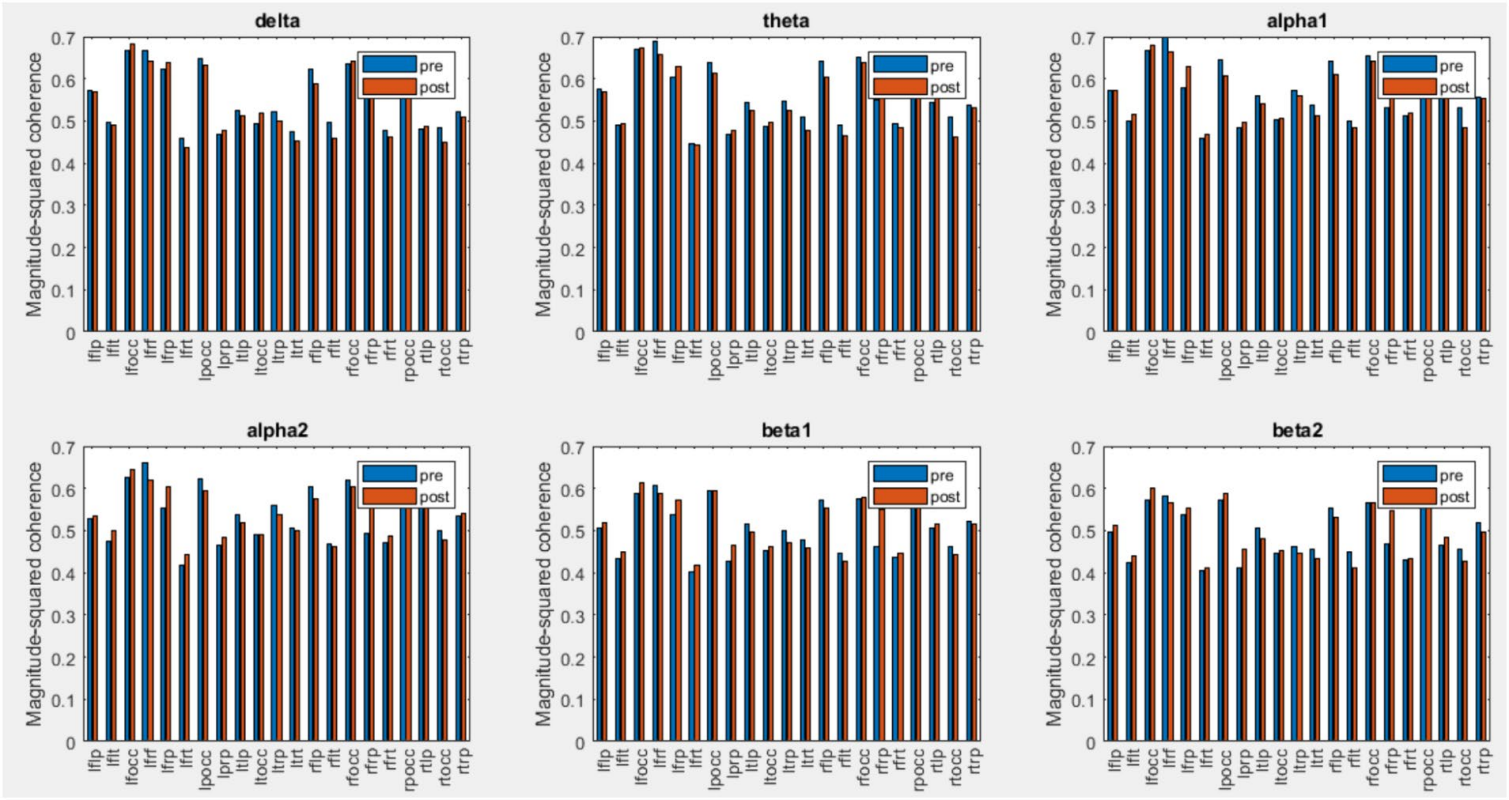
Mean coherence values of all subjects for pre and post-intervention in the control group.

EEG coherence is defined as the spectral correlation between two channel signals used to evaluate temporal synchronization in the frequency domain. EEG coherence of different regions was calculated for both control and chiropractic groups. Figures 9, 10 show the mean coherence values of all subjects for both pre and post-intervention in the chiropractic and control groups. In general, the coherence values of most areas increased in the chiropractic group after the intervention, which was not observed in the control group. Previous research on EEG coherence in healthy individuals and Alzheimer’s patients has shown a reduction in overall cortical connectivity in AD patients (Sankari et al., 2011). In a study by Babiloni et al. (2009), resting state EEG coherence was studied in 28 healthy and 57 aMCI subjects, which aMCI patients were divided into two subgroups: high Cholinergic damage (+MCI) and patients with less damage (−MCI). The results of the study showed that the overall coherence of the alpha1 frequency band was highest in the control group, moderate in the −MCI group and lowest in the +MCI group. The coherence decrease of alpha and beta bands begins in the earliest stages of the disease and is due to the impairment of corticocortical networks (Locatelli et al., 1998). Considering that coherence has increased in most areas, it can be concluded that chiropractic intervention could have a positive effect on the disease process.

## Discussion

One prominent neurophysiological change associated with MCI is a shift in oscillatory EEG power and dominant rhythms from higher to lower frequencies. In this study, the EEG signal power in different frequency bands was analyzed in order to investigate the effects of chiropractic intervention on mild cognitive impairment.

As mentioned, in the chiropractic group, a decrease in power was observed for the delta (0.5–4 Hz) and theta (4–8 Hz) bands, and an increase in power was observed for the beta2 band (25–35 Hz) post the intervention. These results are consistent with previous studies that have shown one of the major effects of MCI and AD is EEG slowing (Dauwels et al., 2010). Mild Cognitive Impairment/AD is associated with an increase of power in low frequencies (delta and theta band, 0.5–8 Hz) and a decrease of power in higher frequencies (alpha and beta, 8–30 Hz) (Dauwels et al., 2010). In MCI patients, the theta band power, especially in the temporal and parietal areas, increases which indicates cognitive impairment. Increasing theta is associated with AHC atrophy as well as with memory deficits, which is a major risk factor for the development of Alzheimer’s disease and MCI (Fauzan, 2015).

Using the SVM method, the highest accuracy was obtained in beta 2, theta, and delta bands, respectively. In the permutation test, the clusters were found in these frequency bands. Previous studies have introduced theta band power as a measure of MCI diagnosis. In MCI patients, the theta band power is higher than in healthy people and has been widely used to distinguish between MCI patients and healthy people (Zappoli et al., 1995; Jelic et al., 1996; Huang et al., 2000). Some studies also show that beta2 band power is correlated with cognitive abilities and is important in cognitive processes. Beta2 band power can predict the progression of Alzheimer’s in the early stages of mild cognitive impairment and could be used as an alternative marker for memory impairment (Kaiser et al., 2017).

Comparing different brain regions, in the delta band the highest accuracy was related to the frontal area. These delta band results of this study are consistent with a study conducted in 2015 by Fauzan (2015) to identify the characteristics of mild cognitive impairment compared to healthy individuals. The mean absolute power amplitude and the mean *z* scores were obtained in different frequency bands. The results of this study show that in MCI patients, the delta power increases in the frontal and temporal areas.

In the alpha1 band, the highest accuracy was related to the occipital and right frontal channels, in the alpha2 band, the highest accuracy was related to the right frontal and temporal areas, in the beta band, the highest accuracy was related to the frontal channels and right temporal lobe and parietal which is higher in beta 2 than beta 1. In previous studies, it has been shown that in MCI patients, the predominance of beta2 is higher in frontal, temporal, and parietal regions. It has also been shown that alpha and beta activity in the right frontal and parietal decreases in the MCI group while resting with open eyes (McBride et al., 2014).

Abnormal changes in EEG coherence between and within the hemisphere in Alzheimer’s patients suggest that the decrease in coherence may be due to the fact that Alzheimer’s causes extensive brain degeneration, leading to fewer neural connections. In addition, lower coherence values may reflect impaired functional connections between and within the hemisphere (Zheng-yan, 2005). Comparison of coherence changes in the control and chiropractic groups shows that the inter-hemispheric coherence changes in all frequency bands are significantly different between the two groups. Research has shown that EEG inter-hemispheric coherence is significantly lower in patients with AD and MCI than in controls (Zheng-yan, 2005; Hadiyoso et al., 2022).

Previous studies have shown the presence of corpus callosum atrophy in patients with AD compared with healthy individuals. In addition, Koeda et al. (1995) showed a decrease in inter-hemispheric coherence in patients with corpus callosum agenesis. Inter-hemispheric coherence shows functional coupling between brain areas, suggesting the corpus callosum plays a key role in EEG synchronization between hemispheres. The present findings indicate that patients with AD do not have proper interaction between the hemispheres, which reflects impaired functional connections between the hemispheres throughout the corpus callosum (Zheng-yan, 2005). The main contribution of hemispheres in the posterior region is due to the effect of cortical fibers that connect the two hemispheres in the anterior cleft, middle and splenium of the corpus callosum, and their integrity is lost in AD patients. Quantitative MRI studies have confirmed a significant reduction in corpus callosum in patients with AD (Locatelli et al., 1998).

Intra-hemispheric coherence changes of the two groups in the left frontal-parietal, right frontal-temporal and right temporal-parietal areas were significant. The results showed that the left frontal-temporal and right frontal-parietal changes in the beta1 and beta2 bands were significantly different in the two groups. In previous studies comparing MCI patients with healthy individuals, reduced intra-hemispheric coherence in the frontal-parietal areas (right hemisphere and left hemisphere) has been reported (Moretti et al., 2008). Intra-hemispheric coherence analysis showed a significant reduction in the connection of the frontal-temporal-central-occipital network, which was found significantly in the pair of electrodes containing FP1. Decreased intrahemispheric coherence in MCI patients is related to the disconnection of cortico-cortical connectivity which connects the temporoparietal, and occipital areas with the frontal areas (Hadiyoso et al., 2022).

Previous research shows that alpha-band coherence is decreased in the temporoparietal region in AD patients compared with the control group. Metabolic activity in the left hemisphere is also affected earlier than in the right hemisphere. Previous EEG studies using power spectrum analysis have shown that in the early stages of the disease, changes can become more pronounced in the left temporoparietal channels (Locatelli et al., 1998). Coherence changes in this area between the two groups were not significant and it seems that one session intervention could not improve the damage in these areas.

These results suggest that chiropractic SM intervention may augment cognitive function in people with MCI. It is possible that chiropractic intervention may even reduce the risk of developing AD or the progression of cognitive impairment, but far more research is needed to define this relationship. Future research could include prospective studies following people with MCI who have regular chiropractic care, and the assessment of cognitive outcomes in people with MCI or AD under chiropractic care. Our study offers insights into the potential impact of chiropractic intervention on MCI. However, several limitations warrant acknowledgment. Individuals with MCI progress at varying rates and respond differently to treatment, potentially influencing observed neurophysiological changes. Additionally, the persistent effects of medication use, notably psychotropic medications for cognitive symptoms, might affect EEG activity and complicate treatment interpretation. Lifestyle factors such as physical activity, diet, and sleep quality, although potentially influential, were not systematically controlled for in our study. Furthermore, the small sample size and lack of a control group restrict the generalizability of our findings and impede causal inferences regarding observed EEG changes. To address these limitations, future research should conduct larger longitudinal studies, considering participant characteristics and lifestyle factors, alongside stringent control measures. Additionally, to evaluate the intervention’s long-term effects, EEG signals should be analyzed over extended post-intervention intervals.

## Conclusion

In this study, a nonparametric cluster-based permutation test and classification with the SVM method were used to investigate changes in EEG signal power in patients with MCI pre- and post-chiropractic or control intervention sessions. A comparison of the relative power of the delta and theta bands showed they decreased after the chiropractic intervention and the power of the beta2 band increased. According to previous studies in the field of MCI, these changes are a sign of improvement in MCI. Using a nonparametric cluster-based permutation test, no significant difference was observed pre- and post-intervention in the control and chiropractic groups. In the chiropractic group, 3 clusters were found in the delta band (*p* values = 0.1506, 0.1673, 0.1712), 2 clusters in the theta band (*p* values = 0.1702, 0.1854), and in the control group one cluster was found in the beta2 band (*p* value = 0.1656).

In order to compare the power changes of the two groups, the SVM method was used for classification. With this method, a high accuracy was obtained for the separation of power changes in control and chiropractic groups in all frequency bands. The highest accuracy was related to the beta2 and theta bands. Previous studies have also reported the importance of these two bands in diagnosing MCI and its progression. The SVM method was also used to investigate which areas were most affected by the intervention.

Coherence was also used to evaluate EEG signal synchrony. The calculation of coherence in different areas showed that, in general, the values of coherence increased in most areas in the chiropractic group post-intervention. Wilcoxon rank sum tests also showed that the interhemispheric coherence changes in all frequency bands are significantly different between the two groups. For intrahemispheric coherence, the changes in the coherence of the two groups were significant in the left frontal-parietal, right frontal-temporal and right temporal-parietal areas. Also, the results of the test showed that the changes in left frontal-temporal and right frontal-parietal coherence in beta1 and beta2 bands are significantly different in the two groups.

A comparison of the results obtained from chiropractic intervention and previous studies shows that chiropractic intervention can have a positive effect on MCI disease and may even reduce the risk of developing AD or the progression of cognitive impairment, but far more research is needed to define this relationship.

## Data availability statement

The data analyzed in this study is subject to the following licenses/restrictions: the data that support the findings of this study are available upon reasonable request. Requests to access these datasets should be directed to IN, imran.niazi@aut.ac.nz.

## Ethics statement

The studies involving humans were approved by Internal Review Board (approval number IRB-67) at the Atta-ur-Rahman School of Applied Biosciences, National University of Sciences and Technology and Riphah College of Rehabilitation Sciences, Islamabad, Pakistan. The studies were conducted in accordance with the local legislation and institutional requirements. The participants provided their written informed consent to participate in this study.

## Author contributions

FZ: Conceptualization, Formal analysis, Investigation, Methodology, Software, Validation, Visualization, Writing – original draft, Writing – review & editing. IN: Conceptualization, Data curation, Funding acquisition, Investigation, Methodology, Resources, Supervision, Writing – review & editing. IA: Data curation, Software, Writing – review & editing. AC: Investigation, Writing – review & editing. JD: Investigation, Writing – review & editing. UG: Investigation, Writing – review & editing. KH: Funding acquisition, Investigation, Resources, Writing – review & editing. HH: Funding acquisition, Investigation, Resources, Writing – review & editing. VS: Conceptualization, Investigation, Methodology, Project administration, Supervision, Validation, Writing – review & editing.

## References

[ref1] Amjad I. Toor H. Niazi I. K. Afzal H. Jochumsen M. Shafique M.. (2018). Therapeutic effects of aerobic exercise on EEG parameters and higher cognitive functions in mild cognitive impairment patients. Int. J. Neurosci. 129:551. doi: 10.1080/00207454.2018.1551894 30929591

[ref2] Babiloni C. Frisoni G. Vecchio F. Lizio R. Pievani M. Geroldi C.. (2009). Global functional coupling of resting EEG rhythms is abnormal in mild cognitive impairment and Alzheimer’s disease: a multicenter EEG study. J. Psychophysiol. 23, 224–234. doi: 10.1027/0269-8803.23.4.224

[ref3] Cooperstein R. Gleberzon B. J. (2004). Technique Systems in Chiropractic: Churchill Livingstone.

[ref4] Cortes C. Vapnik V. (1995). Support vector networks. Mach. Learn. 20, 273–297. doi: 10.1007/BF00994018

[ref5] Czigler B. Csikós D. Hidasi Z. Anna Gaál Z. Csibri É. Kiss É.. (2008). Quantitative EEG in early Alzheimer's disease patients—power spectrum and complexity features. Int. J. Psychophysiol. 68, 75–80. doi: 10.1016/j.ijpsycho.2007.11.002, PMID: 18093675

[ref6] Dauwels J. Vialatte F. Cichocki A. (2010). Diagnosis of Alzheimer’s disease from EEG signals: where are we standing? Curr. Alzheimer Res. 7, 487–505. doi: 10.2174/156720510792231720, PMID: 20455865

[ref7] Ernst M. D. (2004). Permutation methods: a basis for exact inference. Stat. Sci. 19:396. doi: 10.1214/088342304000000396

[ref8] Faghfouri A. Shalchyan V. Toor H. G. Amjad I. Niazi I. K. (2024). A tensor decomposition scheme for EEG-based diagnosis of mild cognitive impairment. Heliyon 10:e26365. doi: 10.1016/j.heliyon.2024.e26365, PMID: 38420472 PMC10901001

[ref9] Fauzan N. (2015). Nur Hurunain Amran, brain dynamics of mild cognitive impairment (MCI) from EEG features. Procedia. Soc. Behav. Sci. 165, 284–290. doi: 10.1016/j.sbspro.2014.12.633

[ref10] Fiscon G. Weitschek E. Cialini A. Felici G. Bertolazzi P. de Salvo S.. (2018). Combining EEG signal processing with supervised methods for Alzheimer’s patients classification. BMC Med. Inf. Decision Making 18:613. doi: 10.1186/s12911-018-0613-y PMC5984382 29855305

[ref11] Gallego-Jutglàa E. Solé-Casalsa J. Vialatteb F. B. Dauwelsc J. Cichocki A. (2015). A thetaband EEG based index for early diagnosis of Alzheimer’s disease running title: EEG based index. J. Alzheimers Dis. 43, 1175–1184. doi: 10.3233/JAD-140468 25147104

[ref12] Gatterman M. (2005). Foundations of chiropractic: Elsevier Mosby.

[ref13] Geldmacher D. S. Whitehouse P. J. (1997). Differential diagnosis of Alzheimer's disease. Neurology 48:e48. doi: 10.1212/WNL.48.5_Suppl_6.2S 9153154

[ref14] Grundman M. Petersen R. C. Ferris S. H. Thomas R. G. Aisen P. S. Bennett D. A.. (2004). Mild cognitive impairment can be distinguished from Alzheimer disease and normal aging for clinical trials. Arch. Neurol. 61, 59–66. doi: 10.1001/archneur.61.1.59, PMID: 14732621

[ref15] Haavik H. Murphy B. (2012). The role of spinal manipulation in addressing disordered sensorimotor integration and altered motor control. J. Electromyogr. Kinesiol. 22, 768–776. doi: 10.1016/j.jelekin.2012.02.012, PMID: 22483612

[ref16] Hadiyoso S. Wijayanto I. Aulia S. (2022). Comparison of resting electroencephalogram coherence in patients with mild cognitive impairment and normal elderly subjects. Int. J. Electr. Comput. Eng. 12, 1558–1564. doi: 10.11591/ijece.v12i2.pp1558-1564

[ref17] Holt K. Niazi I. K. Nedergaard R. W. Duehr J. Amjad I. Shafique M.. (2019). The effects of a single session of chiropractic care on strength, cortical drive, and spinal excitability in stroke patients. Sci. Rep. 9:2673. doi: 10.1038/s41598-019-39577-5, PMID: 30804399 PMC6389925

[ref18] Huang C. Wahlund L. O. Dierks T. Julin P. Winblad B. Jelic V. (2000). Discrimination of Alzheimer’s disease and mild cognitive impairment by equivalent EEG sources: a cross-sectional and longitudinal study. Clin. Neurophysiol. 111, 1961–1967. doi: 10.1016/S1388-2457(00)00454-5, PMID: 11068230

[ref19] Jelic V. Shigeta M. Julin P. (1996). Quantitative electroencephalography power and coherence in Alzheimer’s disease and mild cognitive impairment. Dementia, 314–323, PMID: 8915037 10.1159/000106897

[ref20] Kaiser A. K. Doppelmayr M. Iglseder B. (2017). EEG beta 2 power as surrogate marker for memory impairment: a pilot study. Int. Psychogeriatr. 29, 1515–1523. doi: 10.1017/S1041610217000758, PMID: 28528599

[ref21] Kajihara T. Anwar M. N. Kawasaki M. Mizuno Y. Nakazawa K. Kitajo K. (2015). Neural dynamics in motor preparation: from phase-mediated global computation to amplitude-mediated local computation. NeuroImage 118, 445–455. doi: 10.1016/j.neuroimage.2015.05.032, PMID: 26003857

[ref22] Koeda T. Knyazeva M. Njiokiktjien C. Jonkman E. J. De Sonneville L. Vildavsky V. (1995). The EEG in acallosalchildren. Coherence values in the resting state: left hemisphere compensatory mechanism? Electroencephalogr. Clin. Neurophysiol. 95, 397–407. doi: 10.1016/0013-4694(95)00171-9, PMID: 8536568

[ref23] Langa K. M. Levine D. A. (2014). The diagnosis and Management of Mild Cognitive Impairment: a clinical review. JAMA 312, 2551–2561. doi: 10.1001/jama.2014.13806, PMID: 25514304 PMC4269302

[ref24] Lelic D. Niazi I. K. Holt K. Jochumsen M. Dremstrup K. Yielder P.. (2016). Manipulation of dysfunctional spinal joints affects sensorimotor integration in the prefrontal cortex: a brain source localization study. Neural Plast. 2016, 1–9. doi: 10.1155/2016/3704964, PMID: 27047694 PMC4800094

[ref25] Locatelli T. Cursi M. Liberati D. Franceschi M. Comi G. (1998). EEG coherence in Alzheimer’s disease. Electroencephalogr. Clin. Neurophysiol. 106, 229–237. doi: 10.1016/S0013-4694(97)00129-6 9743281

[ref26] Lopez-Calderon J. Luck S. J. (2014). ERPLAB: an open-source toolbox for the analysis of event-related potentials. Front. Hum. Neurosci. 8:213. doi: 10.3389/fnhum.2014.00213, PMID: 24782741 PMC3995046

[ref27] Luck S. J. (2014). Introduction to the Event-Related Potential: MIT Press Ltd.

[ref28] McBride J. C. Zhao X. Munro N. B. Smith C. D. Jicha G. A. Lee H.. (2014). Spectral and complexity analysis of scalp EEGcharacteristics for mild cognitive impairment and early Alzheimer’s disease. Comput. Methods Prog. Biomed. 114, 153–163. doi: 10.1016/j.cmpb.2014.01.019, PMID: 24598317 PMC4021716

[ref29] Moretti D. V. Frisoni G. B. Pievani M. Rosini S. Geroldi C. (2008). Cerebrovascular disease and hippocampal atrophy are differently linked to functional coupling of brain areas: an EEG coherence study in MCI subjects. J. Alzheimers Dis. 14, 285–299. doi: 10.3233/JAD-2008-14303, PMID: 18599955

[ref30] Navid M. S. Lelic D. Niazi I. K. Holt K. Mark E. B. Drewes A. M.. (2019a). The effects of chiropractic spinal manipulation on central processing of tonic pain—a pilot study using standardized low-resolution brain electromagnetic tomography (sLORETA). Sci. Rep. 9:6925. doi: 10.1038/s41598-019-42984-3, PMID: 31061511 PMC6502880

[ref31] Navid M. S. Niazi I. K. Lelic D. Amjad I. Kumari N. Shafique M.. (2022). Chiropractic spinal adjustment increases the cortical drive to the lower limb muscle in chronic stroke patients. Front. Neurol. 12:747261. doi: 10.3389/fneur.2021.747261, PMID: 35185747 PMC8854235

[ref32] Navid M. S. Niazi I. K. Lelic D. Drewes A. M. Haavik H. (2019b). The effects of Filter’s class, cutoff frequencies, and independent component analysis on the amplitude of somatosensory evoked potentials recorded from healthy volunteers. Sensors 19:112610. doi: 10.3390/s19112610, PMID: 31181744 PMC6603557

[ref33] Navid M. S. Niazi I. K. Lelic D. Nedergaard R. B. Holt K. Amjad I.. (2020). Investigating the effects of chiropractic spinal manipulation on EEG in stroke patients. Brain Sci. 10:50253. doi: 10.3390/brainsci10050253, PMID: 32349288 PMC7288271

[ref34] Niazi I. K. Türker K. S. Flavel S. Kinget M. Duehr J. Haavik H. (2015). Changes in H-reflex and V-waves following spinal manipulation. Exp. Brain Res. 233, 1165–1173. doi: 10.1007/s00221-014-4193-5, PMID: 25579661

[ref35] Oostenveld R. Fries P. Maris E. Schoffelen J. M. (2011). FieldTrip: open source software for advanced analysis of MEG, EEG, and invasive electrophysiological data. Comput. Intell. Neurosci. 2011, 1–9. doi: 10.1155/2011/156869, PMID: 21253357 PMC3021840

[ref36] Oostenveld E. Maris R. (2007). Nonparametric statistical testing of EEG- and MEG-data. J. Neurosci. Methods 164:177. doi: 10.1016/j.jneumeth.2007.03.024 17517438

[ref37] Orange J. B. Ryan E. B. (2000). Alzheimer’s disease and other dementias. Clin. Geriatr. Med. 16, 153–173. doi: 10.1016/S0749-0690(05)70015-X 10723625

[ref38] Sankari Z. Adeli H. Adeli A. (2011). Intrahemispheric, interhemispheric, and distal EEG coherence in Alzheimer's disease. Clin. Neurophysiol. 122, 897–906. doi: 10.1016/j.clinph.2010.09.008, PMID: 21056936

[ref39] Sharma N. Kolekar M. H. Jha K. Kumar Y. (2018). EEG and cognitive biomarkers based mild cognitive impairment diagnosis. IRBM. doi: 10.1016/j.irbm.2018.11.007

[ref40] Steven Waterstone T. Niazi I. K. Navid M. S. Amjad I. Shafique M. Holt K.. (2020). Functional connectivity analysis on resting-state electroencephalography signals following chiropractic spinal manipulation in stroke patients. Brain Sci. 10, 1–18. doi: 10.3390/brainsci10090644 PMC7564276 32957711

[ref41] Triano J. J. Budgell B. Bagnulo A. Roffey B. Bergmann T. Cooperstein R.. (2013). Review of methods used by chiropractors to determine the site for applying manipulation. Chiropr. Man. Ther 21:e36. doi: 10.1186/2045-709X-21-36, PMID: 24499598 PMC4028787

[ref42] Zappoli R. Versari A. Paganini M. Arnetoli G. Muscas G. C. Gangemi P. F.. (1995). Brain electrical activity (quantitative EEG and bit-mapping neurocognitive CNV components) psychometrics and clinical findings in presenile subjects with initial mild cognitive decline or probable Alzheimer-type dementia. Ital. J. Neurol. Sci. 16, 341–376. doi: 10.1007/BF02229172 8626214

[ref43] Zheng-yan J. (2005). Abnormal cortical functional connections in Alzheimer’s disease: analysis of inter- and intra-hemispheric EEG coherence. J. Zhejiang Univ. Sci. 6, 259–264. doi: 10.1631/jzus.2005.B0259 PMC1389734 15754423

